# Chlamydomonas proteases: classification, phylogeny, and molecular mechanisms

**DOI:** 10.1093/jxb/erab383

**Published:** 2021-09-01

**Authors:** Yong Zou, Peter V Bozhkov

**Affiliations:** 1 Department of Molecular Sciences, Uppsala BioCenter, Swedish University of Agricultural Sciences and Linnean Center for Plant Biology, Uppsala, Sweden; 2 Bielefeld University, Germany

**Keywords:** Arabidopsis, autophagy, Chlamydomonas, evolution, green algae, horizontal gene transfer, phylogeny, proteases, protein quality control, proteolytic substrates

## Abstract

Proteases can regulate myriad biochemical pathways by digesting or processing target proteins. While up to 3% of eukaryotic genes encode proteases, only a tiny fraction of proteases are mechanistically understood. Furthermore, most of the current knowledge about proteases is derived from studies of a few model organisms, including *Arabidopsis thaliana* in the case of plants. Proteases in other plant model systems are largely unexplored territory, limiting our mechanistic comprehension of post-translational regulation in plants and hampering integrated understanding of how proteolysis evolved. We argue that the unicellular green alga *Chlamydomonas reinhardtii* has a number of technical and biological advantages for systematic studies of proteases, including reduced complexity of many protease families and ease of cell phenotyping. With this end in view, we share a genome-wide inventory of proteolytic enzymes in Chlamydomonas, compare the protease degradomes of Chlamydomonas and Arabidopsis, and consider the phylogenetic relatedness of Chlamydomonas proteases to major taxonomic groups. Finally, we summarize the current knowledge of the biochemical regulation and physiological roles of proteases in this algal model. We anticipate that our survey will promote and streamline future research on Chlamydomonas proteases, generating new insights into proteolytic mechanisms and the evolution of digestive and limited proteolysis.

## Teaser

Proteases are catabolic or processing enzymes cleaving peptide bonds. *Chlamydomonas reinhardtii* exhibits reduced complexity of many protease families, representing a tractable model for studying these vital but poorly understood enzymes.

## Introduction

Proteolytic enzymes, or proteases, catalyse the cleavage of peptide or isopeptide bonds in proteins. Proteases likely arose early in evolution as merely digestive enzymes necessary for protein degradation and the generation of amino acids in primitive organisms (López-Otín and Bond, 2008). However, apart from being blunt protein distractors, proteases can also act akin to sharp suture scissors by dissecting target polypeptide chains at precise locations into smaller polypeptides, in a process called ‘limited proteolysis’. The result of limited proteolysis is the formation of new post-translationally modified protein species or proteoforms with neo-N- and/or -C-termini (Weng *et al.*, 2019; Liu *et al.*, 2020), which may gain, lose, or show altered biochemical activity compared with their precursor proteins. The actual role and importance of limited proteolysis appears to be greatly underestimated, as it may in fact control myriad biological processes, ranging from seed development and photosynthesis in plants to apoptotic cell death and blood clotting in animals. Acting in a cooperative manner, digestive and limited proteolysis sustain continuous modification of the proteome necessary for normal development and fitness (Minina *et al.*, 2017).

While up to 3% of eukaryotic genes encode proteases that collectively represent the protease degradome of a given organism (Quesada *et al.*, 2008), only a small fraction of proteases are mechanistically understood. This is especially true for plants, where most of the knowledge on proteases is derived from the model organism *Arabidopsis thaliana* (van der Hoorn, 2008). Still, the majority—approximately 86%—of the protease holotypes in Arabidopsis remain uncharacterized biochemically and are known only as sequences (Rawlings, 2020). The monophyletic clade of green plants, consisting of both land plants and green algae, is divided into two evolutionary lineages, the Chlorophyta (chlorophytes) and Streptophyta (streptophytes), that diverged over a billion years ago (Bremer, 1985; Morris *et al.*, 2018). Chlorophyte proteases are largely unknown territory, which limits our mechanistic comprehension of the role of proteolytic mechanisms in plant biology and hampers integrated understanding of how proteolysis evolved.

With these thoughts in mind, we turned our attention to proteolytic enzymes of *Chlamydomonas reinhardtii* (hereafter Chlamydomonas), an ancient unicellular model organism belonging to the Chlorophyta that shares ancestral traits not only with higher plants but also with animals. Thus, Chlamydomonas has been an invaluable reference for studies in the areas of light perception, photosynthesis, chloroplast development, ciliary formation, cell motility, and the cell cycle, among others (Harris, 2001). Whereas the haploid genome of Chlamydomonas expedites functional analysis of genes and proteins of interest, this research is often hindered by the low efficiency of nuclear transgene expression (Salomé and Merchant, 2019). Although the establishment of insertional mutant libraries (Li *et al.*, 2016; Cheng *et al.*, 2017) has aided in some cases, mutants for most genes are still unavailable due to insertion in non-coding regions or a lack of insertion. However, more recent developments of cloning technology (Crozet *et al.*, 2018; Emrich-Mills *et al.*, 2021) and genome editing in Chlamydomonas (Picariello *et al.*, 2020), along with the emerging understanding of transgene silencing mechanisms (Neupert *et al.*, 2020) in Chlamydomonas, should facilitate the functional analysis of various genes of interest in this model alga. 

In multicellular plants, a search for protein substrates whose cleavage by a specific protease would be central to its biological function is significantly hampered by the difficulties in standardizing protein terminomics experiments, due to the complex cellular heterogeneity within the samples used for proteome isolation. Along with the inherent differences in cell physiology and morphology among diverse cell types and tissues that contribute to the variation in the expression, compartmentalization, and activation of a specific protease, there is also responsiveness to both experimental conditions and proteome isolation treatments to consider (Demir *et al.*, 2018; Dissmeyer *et al.*, 2018). In contrast, Chlamydomonas represents a beneficial model system for protease substrate identification by virtue of its limited number of cell types and the possibility of synchronizing the cell cycle and response to external factors within a homogenous cell population.

We argue that the simple unicellular life cycle of Chlamydomonas, coupled with the ease of cell phenotyping and the above-mentioned genetic and technical advantages, provide a powerful paradigm for systematic studies of proteases and proteolytic pathways. Here, we share a genome-wide survey and classification of Chlamydomonas proteases, compare proteases in Chlamydomonas and in Arabidopsis by catalytic type, and classify Chlamydomonas proteases based on their relatedness to major taxonomic groups. Furthermore, we summarize and discuss the available evidence for biochemical regulation and/or physiological roles of proteases in Chlamydomonas.

## Protease degradome of Chlamydomonas versus Arabidopsis

According to the nature of the nucleophile acting during catalysis, proteases are divided into seven major catalytic types: (i) aspartic, (ii) cysteine, (iii) glutamate, (iv) serine, and (v) threonine proteases, as well (vi) metalloproteases and (vii) asparagine peptide lyases. In addition, there are proteases classified as ‘unknown’ and of a ‘mixed catalytic type’ (Rawlings, 2020). While glutamate proteases and asparagine peptide lyases are found only in pathogenic fungi, bacteria, and archaea, the other types of proteases are spread through all domains of life (Jensen *et al.*, 2010; Rawlings *et al.*, 2011).

**Fig. 1.**
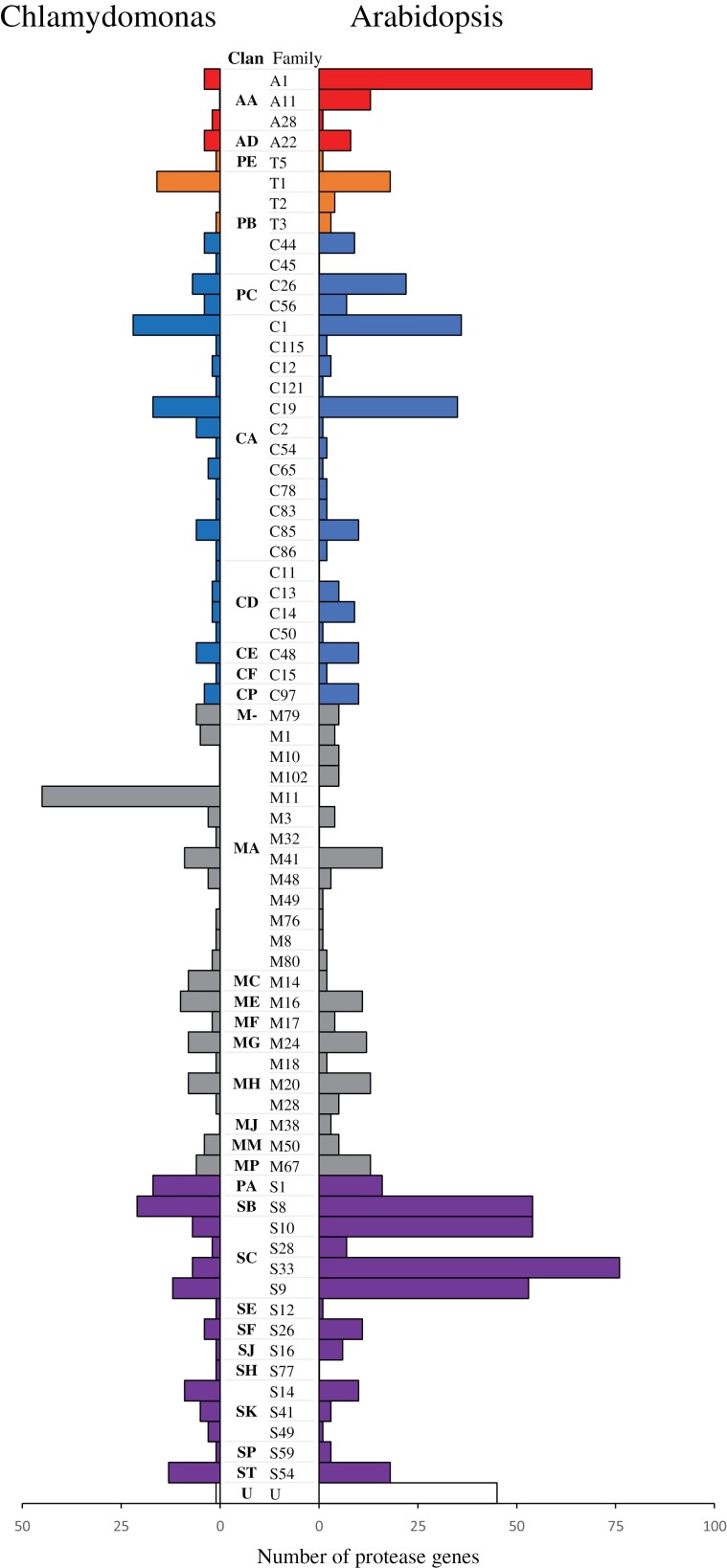
Hierarchical distribution of protease-encoding genes among different clans and families in Chlamydomonas and in Arabidopsis. U, uncharacterized.

We used the major database of proteolytic enzymes, MEROPS, release 12.2 (https://www.ebi.ac.uk/merops/index.shtml) and the Chlamydomonas genome annotation, version 5.5 (https://phytozome.jgi.doe.gov/pz/portal.html#!info?alias=Org_Creinhardtii), to retrieve all protease genes and classify the encoded proteins in comparison with Arabidopsis proteases from both MEROPS and TAIR (https://www.arabidopsis.org/). Altogether, 352 and 764 protease-encoding genes constitute the Chlamydomonas and Arabidopsis degradomes, respectively (Tables S1 and S2 at Zenodo; https://zenodo.org/record/5347045#.YS46It-xWUk, Zou and Bozhkov, 2021).Table 1 shows the distribution of Chlamydomonas and Arabidopsis proteases among catalytic types. One striking difference between the two species is the predominant occurrence of metalloproteases in Chlamydomonas, accounting for ~35% of its degradome (versus only ~15% in Arabidopsis). Notably, more than one-third of the Chlamydomonas metalloproteases (45 out of 124, or ~13% of the whole degradome) belong to the gametolysin family M11, which is absent in Arabidopsis (Fig. 1) and other higher plants. The main role of gametolysin is algal cell-wall degradation to release gametes of both mating types as a necessary prelude to gamete fusion (Matsuda, 2013).

**Table 1.** Number of protease genes in Chlamydomonas and Arabidopsis

	Catalytic type						
Organism	Aspartic	Cysteine	Metallo	Serine	Threonine	Unknown	Total
**Chlamydomonas**	10 (2.8%)	95 (27.0%)	124 (35.2%)	104 (29.5%)	18 (5.1%)	1 (0.3%)	352 (100%)
**Arabidopsis**	91 (11.9%)	172 (22.5%)	117 (15.3%)	313 (41.0%)	26 (3.4%)	48 (5.9%)	764 (100%)

By contrast, there are relatively more aspartic and serine proteases in Arabidopsis than in Chlamydomonas (Table 1), that is, ~12% and ~41% versus ~2.8% and ~30% of the respective degradomes. This is mainly due to the extensive A1 (pepsin-like; 69 homologues) and S33 (prolyl aminopeptidases; 76 homologues) families in Arabidopsis, which are represented by only a few homologues in Chlamydomonas (Fig. 1). In higher plants, the considerable expansion of A1 and S33 proteases may correlate with the specific developmental stages or a plethora of biotic and abiotic stresses that a plant must withstand given its sessile lifestyle (in comparison with motile aquicolous unicellular algae such as Chlamydomonas). For example, a typical pepsin-like A1 protease, constitutive disease resistance 1 (CDR1), elevates the plant resistance to *Pseudomonas syringae* (Xia *et al.*, 2004), whereas a member of the same family, promotion of cell survival 1 (PCS1), is essential for embryogenesis (Ge *et al.*, 2005). Another example is the prolyl aminopeptidase *AtPAP1*, which underlies Arabidopsis tolerance to salt stress and drought (Sun *et al.*, 2013).

Another interesting difference between the degradomes of the two species is the complete absence of certain protease families in one of them. The Chlamydomonas degradome is devoid of aspartic protease family A11 (copia transposon endopeptidase), threonine protease family T2 (glycosylasparaginase precursor), and metalloprotease families M10 (matrixin), M102 (DA1 peptidase), M49 (dipeptidyl-peptidase III, also known as nudix hydrolase 3), and M38 (isoaspartyl dipeptidase), which are represented by one (M49) or more homologues in Arabidopsis (Fig. 1). The presence of the M10 family in Arabidopsis might be associated with defence against biotrophic and necrotrophic pathogens (Zhao *et al.*, 2017), whereas proteases among the M102 family members are known to be involved in organ size control (Vanhaeren *et al.*, 2016; Wang *et al.*, 2017b). In contrast to land plants, the mobile unicellular green algae are capable of evading potential pathogens.

Together with the above-mentioned family M11 (gametolysin), Arabidopsis also lacks the cysteine protease families C11 (clostripain) and C45 (acyl-coenzyme A:6-aminopenicillanic acid acyl-transferase precursor), metalloprotease family M32 (carboxypeptidase), and serine protease family S77 (prohead endopeptidase), which are all present in Chlamydomonas (Fig. 1). However, information about the role of these proteases in the algal life cycle is still lacking.

In contrast to frequently observed gene duplication events, and hence gene redundancy, in higher plants, including Arabidopsis, Chlamydomonas has a simpler genome with much less frequent gene duplications (Merchant *et al.*, 2007). This is also true for protease-encoding genes. Indeed, among 58 protease families present in both Chlamydomonas and Arabidopsis, 41 (~71%) families have more genes per family in Arabidopsis than in Chlamydomonas (Fig. 1). One representative example is family C14, the metacaspases. There are nine metacaspase genes, *AtMC1*–*AtMC9*, in Arabidopsis, which are further split into two structurally distinct types, I and II, represented by three (*AtMC1*, *2*, and *3*, or *AtMCA-Ia*, *b*, and *c* according to new nomenclature) and six (*AtMC4*, *5*, *6*, *7*, *8*, and *9*, or *AtMC-IIa*, *b*, *c*, *d*, *e*, and *f*) homologues, respectively (Tsiatsiani *et al.*, 2011; Minina *et al.*, 2020). Notably, four type II metacaspase genes, *AtMCA-IIa*, *b*, *c*, and *d* (formerly named *AtMC4*, *5*, *6*, and *7*) are products of tandem duplication and are located next to each other within a region of 10.6 kb on Arabidopsis chromosome 1. Additionally, the *AtMCA-IIe* (*AtMC8*) gene situated on the same chromosome is related to the tandem repeat of those four genes by a second internal duplication event (Vercammen *et al.*, 2004). This duplication of type II metacaspase genes in Arabidopsis makes it difficult to decipher the whole spectrum of their mechanistic roles and to distinguish between redundant and individual gene-specific functions. In contrast, the Chlamydomonas genome encodes only one member of each type of metacaspase (CrMCA-I and CrMCA-II; Fig. 1), offering an ideal system for studying the ancestral roles of these proteolytic enzymes.

Interestingly, some protease genes are also found duplicated in Chlamydomonas, suggesting that those gene duplication events likely occurred after divergence from its ancestor shared with higher plants. For example, while Arabidopsis has a single-copy gene encoding Deg1 (for Degradation of periplasmic proteins) protease belonging to the Deg/HtrA (for High temperature requirement A) family (S1), its Chlamydomonas orthologue is represented by three copies (Schuhmann *et al.*, 2012). Based on these facts, we conclude that most of the proteases in Chlamydomonas are encoded by single-copy genes, offering a valuable model for genetic studies.

## Phylogenetic relatedness of Chlamydomonas proteases

As green algae are believed to share a common ancestor with higher plants, and Chlamydomonas shares some features (e.g. cilia) with animals (Merchant *et al.*, 2007), we wondered how such evolutionary versatility affected the phylogenetic relatedness of the Chlamydomonas degradome as a whole. In this analysis, Chlamydomonas protease sequences were used as queries to search against a non-redundant protein database of the National Centre for Biotechnology Information (NCBI). The protein sequences sharing high similarity (E value <10^–10^) were collected for calculating the relative distance through phylogenetic analysis. Based on the closest homologues to algal proteins, all Chlamydomonas proteases were classified into six types: animal, animal and plant, plant, bacterial, green algal, and unclassified (Table S1 at Zenodo). Thus, plant-type proteases are the ones that are most similar to homologues from higher plants, and the same principle determines the ontology of other types, except that animal-and-plant-type proteases are close to the cluster consisting of both animal and plant homologues.

**Fig. 2.**
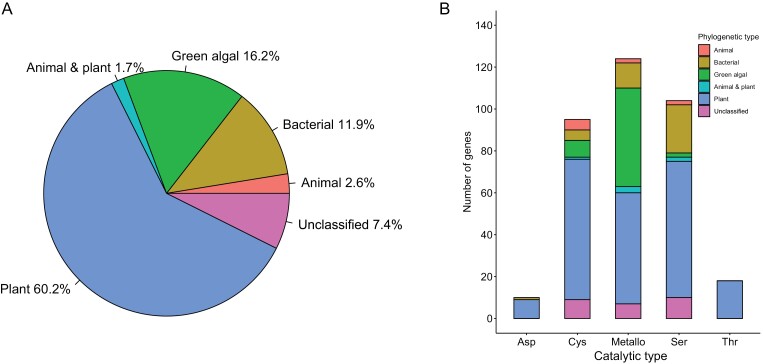
Classification of Chlamydomonas proteases into phylogenetic types. (A) Contribution of different phylogenetic types into the whole Chlamydomonas degradome. (B) Distribution of different phylogenetic types among major catalytic types of Chlamydomonas proteases.

The most abundant (~60%) of Chlamydomonas proteases are of a plant type, followed by green algal type (~16%), and bacterial type (~12%), whereas animal-type along with animal-and-plant-type proteases jointly constitute 4.3% of the Chlamydomonas degradome (Fig. 2A). Interestingly, 7.4% of the Chlamydomonas proteases (26 protein sequences) cannot be ascribed to any phylogenetic type, because they exhibit too high divergence with homologous protein sequences. However, the distribution of all Chlamydomonas proteases into various phylogenetic types presented above does not hold for individual catalytic types. Indeed, all threonine proteases (mostly proteasome subunits) and the vast majority (90%) of aspartic proteases of Chlamydomonas are of the plant type (Fig. 2B). The plant type also predominates among cysteine proteases (~71%), whereas surprisingly large proportions of the metallo- and serine proteases belong to the green algal (~38%) and bacterial (~22%) types, respectively. Finally, the animal type is most frequently found among cysteine proteases (~5.3%; Fig. 2B).

## Biochemistry and physiological role of proteases in Chlamydomonas

### Animal-type proteases

Nine Chlamydomonas proteases are closely related to animal homologues (Table S1 at Zenodo), but none has been systemically studied to date. However, one zinc carboxypeptidase whose homologues are found in ciliated organisms and not in higher plants was originally discovered in the Chlamydomonas ciliary and basal body proteome (gene locus: Cre13.g572850; Li *et al.*, 2004). Despite divergence more than 10^9^ years ago, Chlamydomonas and animals have structurally and functionally similar cilia (Silflow and Lefebvre, 2001). Therefore, the close relationship of proteases involved in cilia formation or function between Chlamydomonas and animals is not surprising. Based on the notion that algal genes might have contributed to the common ancestor of animal genomes via horizontal gene transfer (HGT) (Sun *et al.*, 2010a; Ni *et al.*, 2012), we cannot rule out the possibility that Chlamydomonas genes encoding animal-type proteases were adopted by the animal ancestor and are conserved in extant animal species. Future work on these animal-type proteases will expedite our understanding of their role in cilia formation and activity, or of specific ancestral traits conserved between green algae and animals.

### Plant-type proteases

Plant-type proteases are the most abundant in Chlamydomonas (Fig. 2A), and many are related to the proteasomal and/or organellar protein quality control pathways (Table S1 at Zenodo). Among them are the evolutionarily conserved Filamentation temperature-sensitive H (FtsH), Caseinolytic protease proteolytic subunit (ClpP), DegP, and Lon proteases, which are localized in the chloroplast and/or the mitochondrion and are well known for their role in maintaining organelle homeostasis (Schuhmann and Adamska, 2012; Pinti *et al.*, 2016; Kato and Sakamoto, 2018; Bouchnak and van Wijk, 2021). They digest misfolded proteins and protein aggregates induced by environmental stresses and thus have loose substrate cleavage preference, making substrate specificity screening a challenging task. The ubiquity of these proteases in bacteria and their chloroplastic and/or mitochondrial localization point to their inheritance as a consequence of endosymbiosis, whereas their close intraorganellar co-localization allows them to work cooperatively to degrade common substrates. For example, the cleavage of D1 protein (a core protein in photosystem II) by DegP facilitates subsequent degradation by FtsH under photoinhibitory conditions in Arabidopsis (Kato *et al.*, 2012). Additionally, both ClpP and FtsH degrade the cytochrome *b*_6_*f* complex during nitrogen starvation in Chlamydomonas, although FtsH plays a major role (Majeran *et al.*, 2000; Malnoë *et al.*, 2014; Wei *et al.*, 2014). In the following sections, we consider individual Chlamydomonas proteases with close phylogenetic relationships to homologues from higher plants for which biochemical and/or functional data are available.

#### FtsH1

FtsH is an evolutionarily conserved ATP-dependent zinc-binding metalloprotease (family M41) that is anchored to the chloroplastic or mitochondrial membrane in higher plants and algae. Most prokaryotes encode only one FtsH, whereas eukaryotes generally have several FtsH isoforms, with twelve and six found in Arabidopsis and Chlamydomonas, respectively. Chlamydomonas has close orthologues to all Arabidopsis FtsH proteases except FtsH12 (Table S3 at Zenodo). In addition to the proteolytically active FtsH isoforms, Arabidopsis and Chlamydomonas respectively possess five and three FtsH-like proteins (FtsHi1–FtsHi5 in Arabidopsis), which lost the conserved zinc-binding motif and are presumably catalytically dead (Table S3 at Zenodo). While Arabidopsis FtsH-like proteins are involved in maintaining cellular redox balance (Wang *et al.*, 2018), seedling establishment, and Darwinian fitness in semi-natural conditions (Mishra *et al.*, 2019), the role of their Chlamydomonas homologues remains unknown.

It was shown that Chlamydomonas FtsH1 and FtsH2 were localized at the thylakoid membrane and formed a hetero-oligomer with a molecular mass exceeding 1 MD (Malnoë *et al.*, 2014). The mutation of a conserved arginine residue in the ATPase domain of FtsH1 prevents the integration of the FtsH1/FtsH2 dimer into large supercomplexes and impairs their catalytic activity of degrading photosynthetic membrane protein D1 and cytochrome *b*_6_*f* complex (Malnoë *et al.*, 2014; Table 2). In addition, the dimerization and oligomerization of FtsH1 and FtsH2 are redox-regulated at the intermolecular disulfide bridges formed between as yet unknown cysteine residues (Wang *et al.*, 2017a). Research in higher plants revealed that the FtsH supercomplex in thylakoid membranes has a major role in photosystem II repair and the biosynthesis of photosystem I (reviewed in Kato and Sakamoto, 2018). However, studies of FtsH proteases in Chlamydomonas extend their role to degrading cytochrome *b*_6_*f* complex (Malnoë *et al.*, 2014), in this way connecting carbon fixation and ATP synthesis.

**Table 2.** Proteases functionally characterized in Chlamydomonas

Protease	Gene locus	Clan	Family	Biological role	Substrates	Functional analysis	References
**ATG4**	Cre12.g510100	CA	C54	Autophagy	ATG8	Chemical modulation	Pérez-Pérez *et al.*, 2016
**ClpP1**	CreCp.g001500	SK	S14	Chloroplast protein homeostasis	Cytochrome *b*_6_*f* complex, ATP synthase, Rubisco	Reverse genetics	Ramundo *et al.*, 2014; Majeran et al., 2019, 2000
**DegP1C**	Cre12.g498500	PA	S1	Chloroplast protein homeostasis	D1	Reverse genetics	Schuhmann *et al.*, 2012; Theis *et al.*, 2019
**FtsH1** **FtsH2**	Cre12.g485800 Cre17.g720050	MA	M41	Chloroplast protein homeostasis	D1, cytochrome *b*_6_*f* complex	Reverse genetics	Malnoë *et al.*, 2014; Wang *et al.*, 2017a
**Gametolysin**	Cre17.g718500	MA	M11	Gamete cell wall lysis	Proline- and hydroxyproline-rich proteins	Chemical modulation	Matsuda *et al.*, 1990; Matsuda, 2013
**SMT7**	Cre16.g692600	CE	C48	Cell division	SUMOylated RPL30	Reverse genetics	Lin *et al.*, 2020
**Sporangin**	Cre01.g049950	SB	S8	Sporangial cell wall lysis	Sporangial cell wall proteins	Chemical modulation	Matsuda *et al.*, 1995; Kubo *et al.*, 2009

#### ClpP1

The Clp complex shares both functional and structural similarities with the proteasome (Yu and Houry, 2007). The complex in Arabidopsis consists of three components: proteases, chaperones, and modulators (Peltier *et al.*, 2004). The proteases include six catalytically active ClpPs (AtClpP1–6) and four regulatory ClpP-like proteins (ClpRs; AtClpR1–4) lacking the catalytic residues for peptide bond hydrolysis, and all ten of these proteins are classified into family S14. Among them, AtClpP2 forms a single homotetradecameric complex in the mitochondrion, while the others are components of the plastidic ClpP protease core (Peltier *et al.*, 2004), which is essential for chloroplast development at embryonic and post-embryonic stages. Moreover, mutations of several *Clp* genes (*AtClpP3*, *AtClpP4, AtClpP5, AtClpR2*, and *AtClpR4*) in Arabidopsis are lethal at the embryo or seedling stage (summarized in Olinares *et al.*, 2011).

Based on the presence of a conserved Ser-His-Asp catalytic triad, sequence similarity, and phylogeny (Fig. S1 at Zenodo), four *ClpP* genes (*CrClpP1*, *CrClpP2*, *CrClpP3*, and *CrClpP5*) and four *ClpR* genes (*CrClpR1–4*) are present in the Chlamydomonas genome (Table S3 at Zenodo). In addition, one Clp homologue, CrClpR6 (gene locus Cre06.g299650), clusters closely with AtClpP6, despite missing a catalytic histidine (Fig. S1 at Zenodo) and hence being predicted to lack enzymatic activity (Majeran *et al.*, 2005). Although ClpP family members are highly conserved among species (Yu and Houry, 2007), CrClpP1, as well as homologues from close Chlorophyceae relatives, contain a 30 kD insertion that is absent in ClpP homologues from other organisms (Huang *et al.*, 1994; Derrien *et al.*, 2009). This insertion can be cleaved to generate a short ClpP1 isoform (Majeran *et al.*, 2005), albeit both the long and short CrClpP1 are found within the ClpP protease core (Huang *et al.*, 1994; Derrien *et al.*, 2009).

A failure to obtain ClpP mutants in green plants suggests an indispensable role for this protease complex. The function of CrClpP1 was investigated by taking advantage of a repressible chloroplast gene expression system in Chlamydomonas (Ramundo *et al.*, 2013). It has been found that gradual depletion of CrClpP1 impairs chloroplast morphology and induces autophagy via an unknown retrograde signalling mechanism (Ramundo *et al.*, 2014; Ramundo and Rochaix, 2014). Transcriptomic and proteomic analyses of *crclp1*-deficient strains revealed 16 potential chloroplastic substrates (Ramundo *et al.*, 2014) but failed to detect components of the cytochrome *b*_6_*f* complex (Majeran *et al.*, 2000) and two additional recently identified substrates: small subunits of Rubisco and non-assembled subunits of ATP synthase (Majeran *et al.*, 2019; Table 2).

#### DegP1C

The DegP/HtrA family (S1) members contain an N-terminal trypsin-type protease domain and at least one C-terminal PDZ (from Post synaptic density protein, Drosophila disc large tumor suppressor, and Zonula occludens-1) domain (Clausen *et al.*, 2002). The Arabidopsis genome encodes 16 DegP/HtrA family members (DegP1–DegP16) with different intracellular localizations (Table S3 at Zenodo). The ‘core set’ of DegP proteases typical for green plants includes thylakoid lumen-localized DegP1, DegP5, and DegP8, chloroplast stroma-localized DegP2 and DegP7, nucleolus-localized DegP9, peroxisome-localized DegP15, and mitochondrion-localized DegP10 (Schuhmann *et al.*, 2012). Interestingly, chloroplast-localized DegP proteases are involved in the photosystem II repair cycle by degrading D1 protein at different sites (Kapri-Pardes *et al.*, 2007; Sun et al., 2007, 2010b; Knopf and Adam, 2018).

The Chlamydomonas genome encodes 15 DegP proteins, including duplicated DegP1 isoforms (DegP1A, B, and C). *In vitro* data show that the proteolytic activity of Arabidopsis DegP1 and Chlamydomonas DegP1C is redox-regulated and pH-dependent (Chassin *et al.*, 2002; Ströher and Dietz, 2008; Kley *et al.*, 2011; Theis *et al.*, 2019), and that DegP1C activity is also regulated by temperature (Theis *et al.*, 2019). In Arabidopsis, high light and heat stress were shown to enhance the transcript and protein levels of DegP1, respectively (Itzhaki *et al.*, 1998; Sinvany-Villalobo *et al.*, 2004). Likewise, Chlamydomonas DegP1C is induced at both the protein and the transcript level by various stresses, such as sulphur and phosphorus starvation and heat stress (Zhang *et al.*, 2004; Moseley *et al.*, 2006; Schroda *et al.*, 2015; Theis *et al.*, 2019). In Arabidopsis, *DegP1* knockdown lines exhibit suppressed growth, earlier flowering, and high photoinhibition sensitivity (Kapri-Pardes *et al.*, 2007); however, a Chlamydomonas *degp1c* knockout mutant shows no discernible phenotype under both normal and stress (high light or heat) conditions (Theis *et al.*, 2019), pointing to functional redundancy of the DegP1 isoforms in the alga. Quantitative shotgun proteomics have identified 115 proteins, which are significantly enriched in the Chlamydomonas *degp1c* mutant compared with the wild type, indicating that they are potential substrates of DegP1C (Theis *et al.*, 2019). Among them are all subunits of the cytochrome *b*_6_*f* complex (Table 2), which are known substrates of FtsH and ClpP proteases (Majeran *et al.*, 2000; Malnoë *et al.*, 2014; Wei *et al.*, 2014).

#### Lon

The Lon protease is named after the long filament phenotype of a corresponding bacterial mutant. Prokaryotes and unicellular eukaryotes have a single, mitochondrion-localized Lon, whereas multicellular eukaryotes often contain an extra peroxisomal copy (Tsitsekian *et al.*, 2019). There are four genes encoding Lon isoforms (AtLon1–4) in Arabidopsis. AtLon1 plays an essential role in mitochondrial protein homeostasis (Li *et al.*, 2017), and its deficiency leads to post-embryonic growth retardation, aberrant mitochondrial morphology, and impaired respiration (Rigas *et al.*, 2009; Solheim *et al.*, 2012). The peroxisome-localized AtLon2 facilitates protein import into the matrix and matrix protein degradation to sustain peroxisomal function (Lingard and Bartel, 2009; Farmer *et al.*, 2013). Accordingly, a deficiency of AtLon2 inhibits growth and enhances the autophagic clearance of peroxisomes (pexophagy; Farmer *et al.*, 2013; Bartel *et al.*, 2014). The functions of AtLon3 and AtLon4 remain unknown.

Chlamydomonas has a single mitochondrial Lon (Cre06.g281350), which is absent from the MEROPS database at the time of this writing. The expression level of *CrLon* is high during light periods and low at night, suggesting a potential light-dependent or circadian mechanism (Zones *et al.*, 2015; Fig. S2 at Zenodo). In contrast, the expression of all four *AtLon1–4* genes in Arabidopsis is relatively stable during a 12 h light:12 h dark cycle (Ng *et al.*, 2019; Fig. S2 at Zenodo). While no genetic evidence for *CrLon* is available, the above data point to divergent regulatory mechanisms of *Lon* expression in the chlorophyte and angiosperm lineages.

#### Chlapsin

Chlapsin (Cre04.g226850) is the only studied aspartic-type protease from Chlamydomonas. It belongs to family A1, and features a modified catalytic motif DTG/DSG in contrast to the DTG/DTG typical for A1 family members from higher plants (Almeida *et al.*, 2012). Most aspartic proteases from higher plants are localized in the lytic vacuoles and apoplast, and are active under acidic conditions (Simões and Faro, 2004). While chlapsin shares a requirement for low pH with the higher-plant homologues, it is localized to the chloroplast (Almeida *et al.*, 2012), presumably reflecting different functions. Notably, overexpression of the chlapsin orthologue AtAPA1 confers drought resistance in Arabidopsis (Sebastián *et al.*, 2020), but whether this observation might be relevant to the response of Chlamydomonas living in a humid environment to osmotic stress (Meijer *et al.*, 2001) is still unknown.

#### ATG4

Autophagy is a conserved catabolic process in eukaryotes for recycling cytoplasmic components and membrane-bound organelles. This process is regulated by ATG (for AuTophaGy-related) proteins and entails the fusion of double-membrane vesicles (termed autophagosomes) that can carry various types of cargo, with lysosomes in animals or lytic vacuoles in fungi and plants (Klionsky *et al.*, 2021; Yoshimoto and Ohsumi, 2018). Formation of the autophagosome is reliant on the ATG8 and ATG12 ubiquitin-like conjugation systems (Mizushima, 2020). Cysteine protease ATG4 (family C54), the only proteolytic enzyme among all known ATG proteins, cleaves the nascent ATG8 at a conserved C-terminal glycine to allow the subsequent conjugation of ATG8 to phosphatidylethanolamine, a necessary step for anchoring ATG8 in the autophagosomal membrane. In addition, ATG4 possesses delipidating activity and can cleave the amide bond between phosphatidylethanolamine and ATG8 for the deconjugation and potential reuse of ATG8 (Abreu *et al.*, 2017; Kauffman *et al.*, 2018).

In Arabidopsis, there are two ATG4 isoforms, ATG4a and ATG4b, with the former isoform being more efficient in processing all nine ATG8 protein family members (Woo *et al.*, 2014). Similar to mammalian and yeast ATG4 (Pérez-Pérez *et al.*, 2014; Scherz-Shouval *et al.*, 2007), the proteolytic activity of both Arabidopsis ATG4 isoforms was shown to be reversibly inhibited by oxidation (Woo *et al.*, 2014). However, the molecular details of this redox regulation and its role in the context of plant autophagy remained unknown. In fact, Chlamydomonas research carried out in the laboratory of José Luis Crespo was pivotal for advancing mechanistic understanding of the redox regulation of ATG4, as a part of the autophagy process (for a recent review, see Pérez-Pérez *et al.*, 2021). In particular, it has been found that a single Chlamydomonas ATG4 shares a conserved cysteine (Cys-400), which is absent in the homologues from higher plants, with the yeast ATG4 for redox regulation (Pérez-Pérez *et al.*, 2016). Thus, the proteolytic activity of ATG4 is tightly associated with the cellular environment, with ATG4 shuttling between three major reversible states depending on the redox conditions and the intracellular redox state. In the reduced condition, ATG4 predominantly exists as a catalytically active monomer. The increase in the intracellular redox potential induces the formation of a single disulfide bond at the regulatory Cys-400 residue that inhibits the activity of monomeric ATG4. As the redox potential increases further, the ATG4 monomers oligomerize to form aggregates devoid of proteolytic activity (Pérez-Pérez *et al.*, 2016). Similarly, in animals, ATG4 oligomerization is also triggered by reactive oxygen species production during LC3 (ATG8 orthologue in animals)-associated phagocytosis; the aggregation of ATG4 proteins inhibits their LC3-delipidation activity (Ligeon *et al.*, 2021). In summary, environmental signals affect the intracellular redox changes, leading to the variation of ATG4 conformation and activity. The level of ATG8 and its lipidated form, the main substrates of ATG4, vary accordingly, thus relaying autophagic (in plants) and phagocytic (in animals) activities.

#### SMT7

Protein SUMOylation, the process of covalent conjugation of small ubiquitin-like modifier (SUMO) proteins to target proteins, is a rapid, dynamic, and reversible post-translational mechanism regulating fundamental cellular processes such as cell proliferation and death, as well as stress responses (Zhao, 2018). SUMO proteases catalyse two major cleavage reactions: (i) C-terminal processing of the neo-synthesized immature SUMO for its maturation, and (ii) deconjugation of SUMO from the SUMOylated proteins for retrieval of a free SUMO (Hickey *et al.*, 2012; Yates *et al.*, 2016).

A recent study by Lin *et al.* (2020) has made an interesting attempt to connect SUMOylation to cell-cycle control in Chlamydomonas. Chlamydomonas normally performs cell enlargement during a prolonged G1 phase before undergoing multiple cell fission events in a series of rapid alternating S phases and mitoses (S/M), to produce uniform daughter cells (Cross and Umen, 2015). Mating type locus 3 (MAT3), a retinoblastoma protein, controls the Chlamydomonas cell size for commitment to cell division (Umen and Goodenough, 2001). Correspondingly, *mat3* mutants initiate cell fission at a smaller size and exhibit extra rounds of S/M, resulting in much smaller cell size than wild type (Umen and Goodenough, 2001). Genetic suppressor screens of the *mat3* phenotype identified MAT3 7 (SMT7; Fang and Umen, 2008), one of the six SUMO proteases (family C48) encoded by the Chlamydomonas genome.

There is evidence that catalytic inactivation of proteolytic enzymes by amino acid substitution can slow down dissociation from the substrates without effecting their binding, and thus can be used as a substrate-trapping method (Elmore *et al.*, 2011). Lin *et al.* (2020) took advantage of this method to search for SMT7 substrates by overexpressing the catalytically inactive SMT7^C928A^ in *smt7mat3* double mutants, leading to the identification of ribosomal protein L30 (RPL30) (Lin *et al.*, 2020; Table 2). The authors demonstrated that SMT7 could cleave the SUMO off from the SUMOylated RPL30. The SMT7-dependent deSUMOylation of RPL30 promotes cell division, contributing to the small size of *mat3* mutants (Lin *et al.*, 2020), and provides an explanation for the suppression of the small-cell-size defect of *mat3* mutants by the SMT7 deficiency (Fang and Umen, 2008). However, the molecular mechanism connecting the SUMOylation and deSUMOylation of RPL30 with cell-cycle control remains unknown.

### Green-algal-type and bacterial-type proteases

**Fig. 3.**
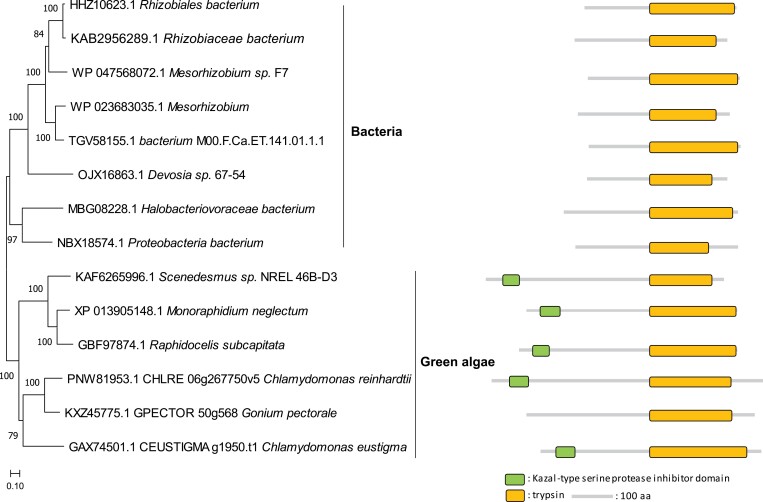
Phylogenetic relationship and domain composition of a trypsin-like peptidase (Cre06.g267750) and related proteases from bacteria and green algae. The search was made against the non-redundant (nr) database from NCBI and protein sequences were used for further alignment and phylogeny analysis. The phylogenetic and domain analyses were performed using MEGA X with the neighbour-joining method (Kumar *et al.*, 2018) and Pfam (El-Gebali *et al.*, 2019), respectively. The scale bar indicates the number of amino acid substitutions per site.

About 16% of all proteases in Chlamydomonas are found exclusively in the green algal lineage (Fig. 2A) and are probably the products of *de novo* originated genes. Most of the green-algal-type proteases are gametolysin isoforms with conserved zinc-binding sites (Fig. S3 at Zenodo). The remaining 12% of Chlamydomonas proteases have a close relationship with homologues from bacteria (Fig. 2A), pointing to HGT that occurred after the separation between Chlamydomonas and higher plants. Curiously, one Chlamydomonas serine protease from the S1 family (gene locus Cre06.g267750; V8 proteinase) contains a C-terminal trypsin-like domain and an N-terminal Kazal-type serine protease inhibitor domain, while its closest homologues from bacteria contain only the trypsin-like domain (Fig. 3). Interestingly, the Chlamydomonas genome also encodes Kazal-type serine protease inhibitors, small proteins with one or more Kazal domains, each being 40–60 amino acids in length. However, it remains to be seen whether these inhibitors interact with and block the proteolytic activity of the V8 proteinase.

Systematic research on copper regulation in Chlamydomonas identified a Regulator of Sigma-E Protease (RSEP1), closely related to RseP, which is a bacterial membrane metalloprotease involved in transmembrane signalling (Castruita *et al.*, 2011). RSEP1 contains a conserved HExxH motif embedded in a transmembrane helix for metal ion binding. Eukaryotic RSEP1 proteases are found exclusively in Chlamydomonadales, suggesting an early HGT from bacteria. In Chlamydomonas, the expression of *RSEP1* is induced by copper depletion in a global copper-sensing transcription factor CRR1-dependent manner (Castruita *et al.*, 2011). It has been suggested that chloroplast-localized RESP1 degrades plastocyanin to release the copper for survival in a copper-deficient environment (Castruita *et al.*, 2011; Kropat *et al.*, 2015), but the genetic evidence for such a role of RESP1 is still missing.

#### Gametolysin and sporangin

Two cell-wall-digesting proteolytic enzymes, bacterial-type sporangin (family S8 of the SB clan) and green-algal-type gametolysin (family M11 of the MA clan), serve important roles in the accomplishment of Chlamydomonas asexual and sexual cycles, respectively. While sporangins from green algae are closely related to bacterial serine proteases (Fig. S4 at Zenodo) and therefore might be a result of an HGT event, gametolysins are found exclusively in Chlamydomonas and its close but multicellular relative *Volvox carteri*.

**Fig. 4.**
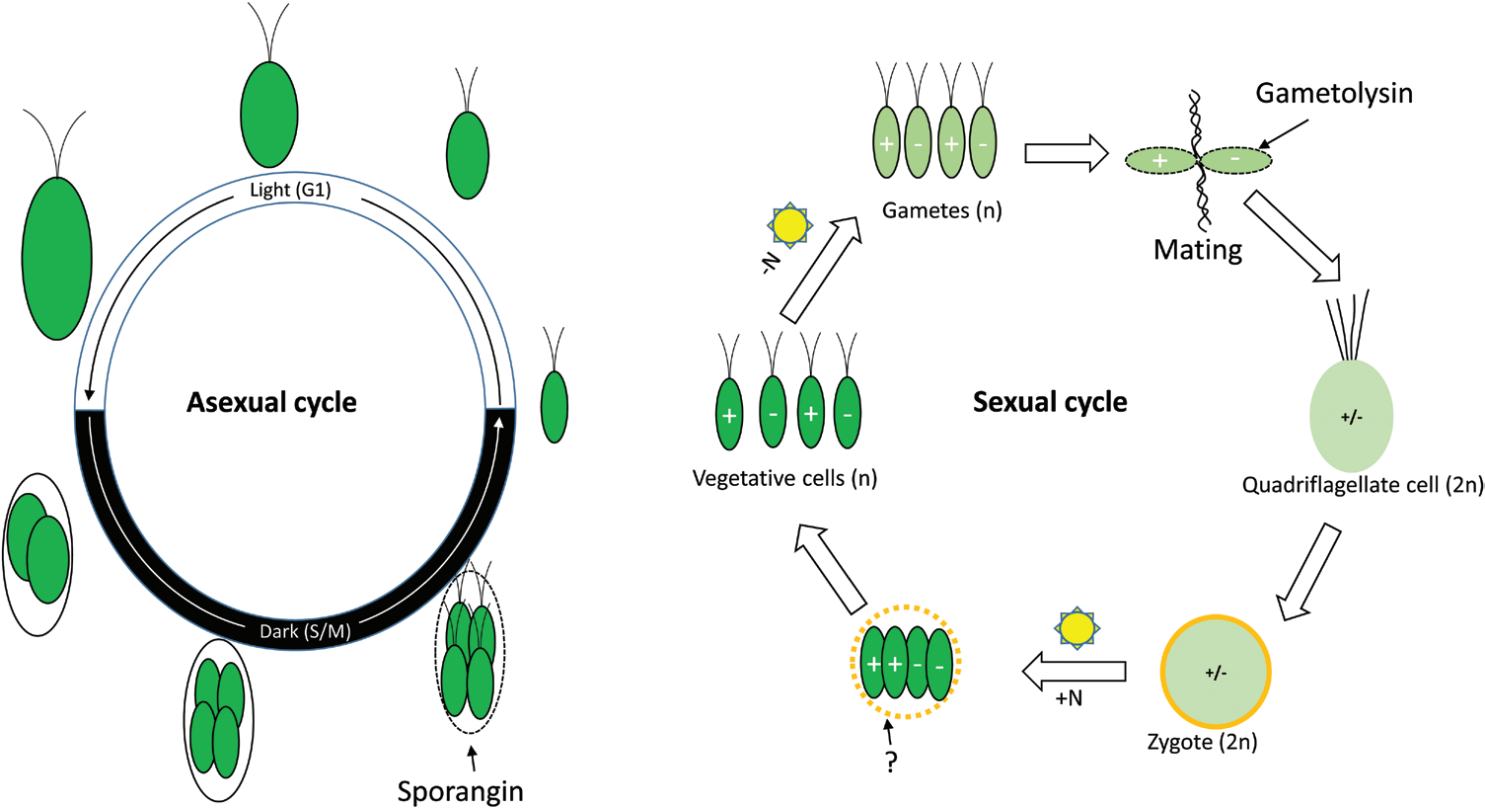
Schematic illustration of the roles of sporangin and gametolysin in Chlamydomonas. Depending on the environmental conditions, Chlamydomonas can undergo either asexual (left) or sexual (right) reproduction cycles. The asexual cycle requires repeated alternating light/dark periods and replete nutrients, and is composed of two broad phases: the light-dependent cell growth (or G1) phase and the dark-dependent cell division (or S phases and mitoses, S/M) phase. Following S/M, daughter vegetative cells hatch out of the mother cell wall digested by sporangin (depicted by the black dotted oval). Under adverse conditions, such as nitrogen deprivation (-N) under light, the vegetative cells transform into gametes of mating type plus or minus. Two gametes of different mating types fuse to form a quadriflagellate cell in a process requiring the proteolytic activity of gametolysin, which removes gametic cell walls (depicted by the black dotted ovals). The quadriflagellate cell loses its cilia and becomes a mature zygote with a thick cell wall. Repletion of nitrogen (+N) induces the zygote to undergo meiosis, which will generate four haploid cells, two of each mating type. Whether proteases are involved in the liberation of haploid cells from the zygote cell wall remains unknown.

Under favourable growth conditions, Chlamydomonas reproduces itself asexually by fission, when a single mother cell undergoes one to three divisions, producing two, four, or eight daughter cells encircled by the mother (sporangial) cell wall (Fig. 4). Sporangin is capable of breaking down the sporangial cell wall during hatching, and its expression is specifically induced during the S/M phase of the asexual cell cycle (Kubo *et al.*, 2009). Under adverse conditions, Chlamydomonas initiates its sexual life cycle, in which haploid gametes of plus and minus mating types fuse to generate a diploid zygote that will divide into four vegetative cells when growth conditions return to normal. Gametolysin is secreted to digest and remove the gamete cell wall, thus allowing mating to commence (Fig. 4). It is noteworthy that gametolysin also exhibits lytic activity towards the cell walls of vegetative and sporangial cells (summarized in Matsuda, 2013).

While there are only six genes encoding sporangin isoforms (Fig. S4 at Zenodo), the gametolysin family contains more than 40 members (Fig. 1). In Chlamydomonas, the sexual cycle represents not only a mode of reproduction, but also a strategy of overcoming adverse conditions (Suzuki and Johnson, 2002). Therefore, a large number of gametolysins present in Chlamydomonas might provide a robust mechanism facilitating cell-wall lysis not only in the secreting gametic cell, but also in surrounding cells, especially those with the opposite mating type, and in this way sustain algal fitness and survival under adverse conditions.

One representative of each of the gametolysin and sporangin families has been studied biochemically in more detail, and they were found to exhibit distinct P1 substrate specificity, potentially accounting for distinct target cell-wall specificity (Table 2). While gametolysin cleaves peptide bonds preferentially after hydrophobic residues (Matsuda *et al.*, 1990), sporangin, similar to many other serine proteases, requires arginine or lysine at the P1 position (Matsuda *et al.*, 1995). Gametolysin is a zinc-binding metalloprotease and requires metal ions for catalysis; accordingly, metal-binding chelators such as EDTA inhibit gametolysin activity (Matsuda *et al.*, 1990). Although metal ions are presumably not required for the catalytic activity of serine proteases, they might be required for stabilization of the active conformation of the proteases. In line with this notion, it has been shown that EDTA inhibits the proteolytic activity of sporangin (Matsuda *et al.*, 1995).

## Concluding remarks

Chlamydomonas has emerged as a unique model representing an early diverged ancestor of higher plants and maintaining some features of the last eukaryotic common ancestor, such as cilia. Thus, Chlamydomonas offers a great evolutionary perspective for research on the mechanisms of cell motility and multicellular pattern formation, besides providing a comparable platform for studying metabolic and signalling pathways operating in higher plants. Since these mechanisms and pathways involve proteolytic regulation, and many proteases of Chlamydomonas are encoded by single-copy genes, the value of this model organism for studying proteolysis is difficult to overestimate.

In this review, we have attempted to offer a comprehensive survey of the protease degradome in Chlamydomonas that may be useful for conceiving future research endeavours. The functional studies performed thus far (Table 2) cover just a tiny fraction of Chlamydomonas proteases whose functions still need to be linked to specific proteolytic events. Overall, protease substrates and proteome modifications caused by proteolysis in Chlamydomonas remain unknown. This lack of knowledge calls for future studies of the Chlamydomonas protease degradome, which should be facilitated by the recent advent of N-terminomics (Luo *et al.*, 2019), positional scanning substrate combinatorial library (PS-SCL) screening (Poręba *et al.*, 2014), and methods for live imaging of protease activity (Fernández-Fernández *et al.*, 2019).

## Supplementary Material

erab383_suppl_Supplementary_Figures_S1_S4

erab383_suppl_Supplementary_Tables_S1_S3

## Data Availability

The data that support the findings of this study are openly available in Zenodo at https://zenodo.org/record/5347045#.YS46It-xWUk; Zou and Bozhkov (2021).
