# Supplementary material for: Chlamydomonas proteases: classification, phylogeny, and molecular mechanisms
Source: J Exp Bot. 2021 Sep 1;72(22):7680–93. doi: 10.1093/jxb/erab383 (PMC8643629; doi:10.1093/jxb/erab383)
Supplement: erab383_suppl_Supplementary_Figures_S1_S4 [file erab383_suppl_supplementary_figures_s1_s4.pdf]

Supplementary material for

Chlamydomonas proteases: classification, phylogeny and molecular mechanisms

Yong Zou and Peter V. Bozhkov

Department of Molecular Sciences, Uppsala BioCenter, Swedish University of Agricultural Sciences and Linnean Center for Plant Biology, Uppsala, Sweden

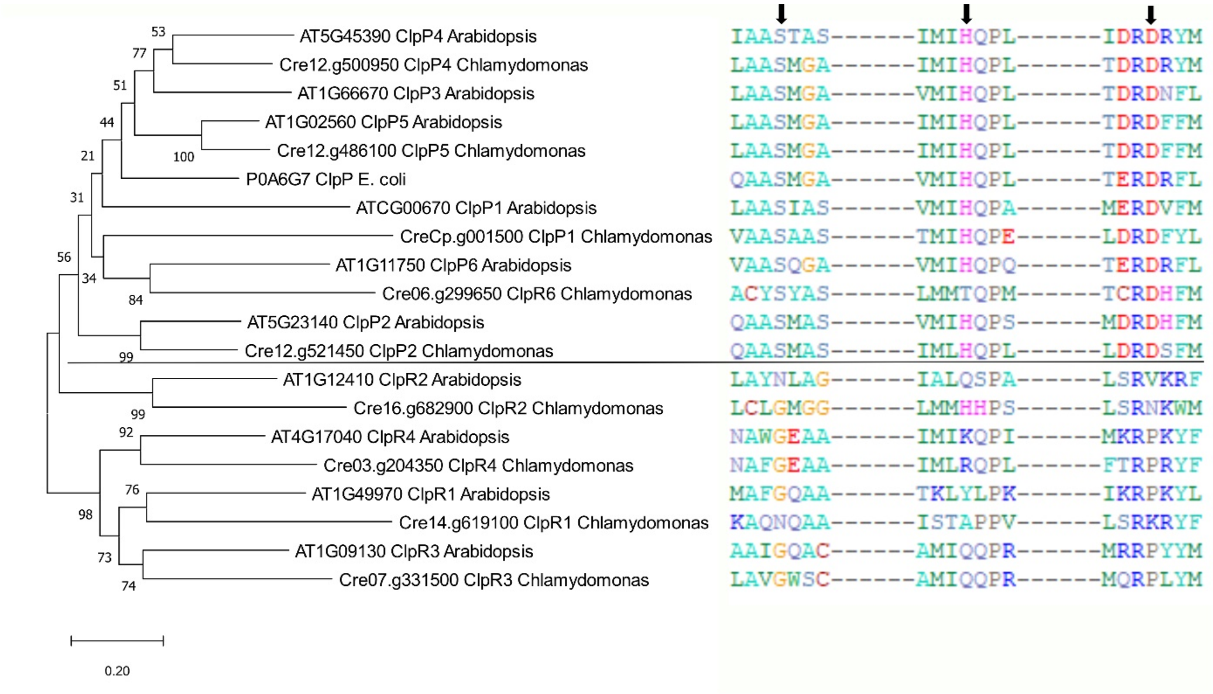

Supplemental Fig. S1 The phylogeny and sequence comparison of Clp homologues in Chlamydomonas and Arabidopsis. The Clp protease from *E. coli* is also included. The phylogenetic analysis was performed as in Fig. 3. The Clp proteins above the horizontal line possess three conserved catalytic residues (indicated by arrows) and are presumably proteolytically active. The only exception is Chlamydomonas ClpR6 lacking catalytic histidine. The Clp proteins below the line are presumably catalytically inactive.

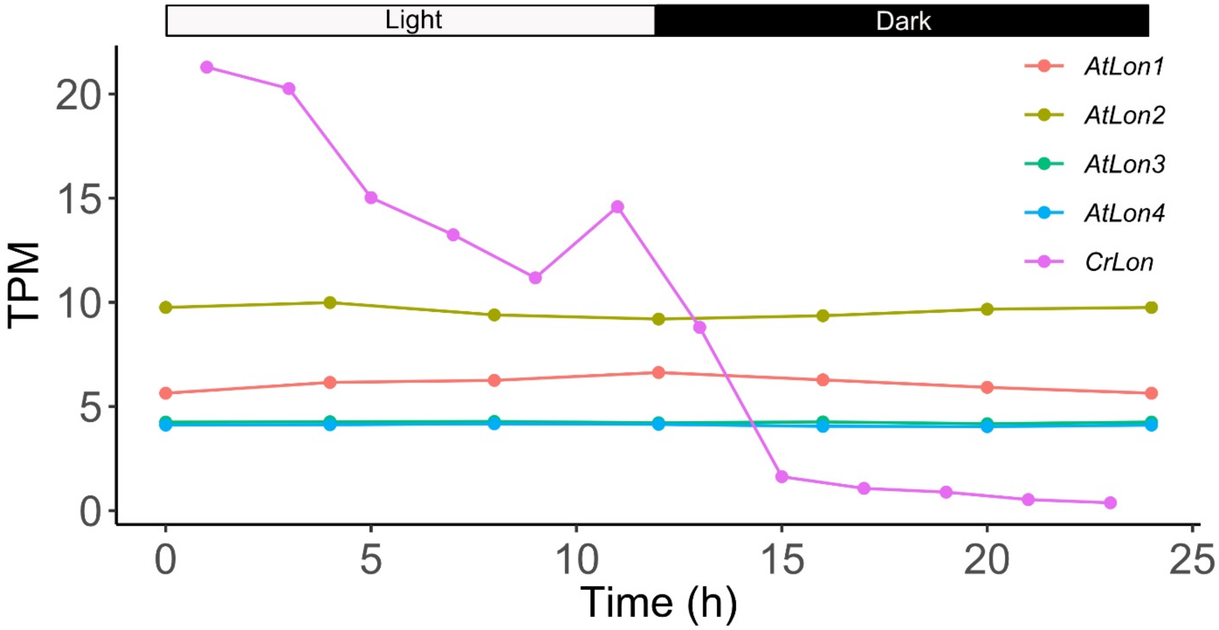

Supplemental Fig. S2. The expression profiles of *Lon* genes in Chlamydomonas and Arabidopsis under diurnal cycle. Data are obtained from Ng *et al.* (2019). The light and dark periods are indicated. TPM, Transcripts Per Kilobase Million.

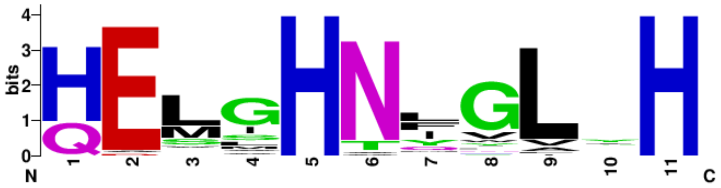

Supplemental Fig. S3 The weblog of conserved zinc binding sites of gametolysin constructed using the WebLogo online software (Crooks *et al.*, 2004). Height of letters reflects the relative frequency of the corresponding amino acids at that position.

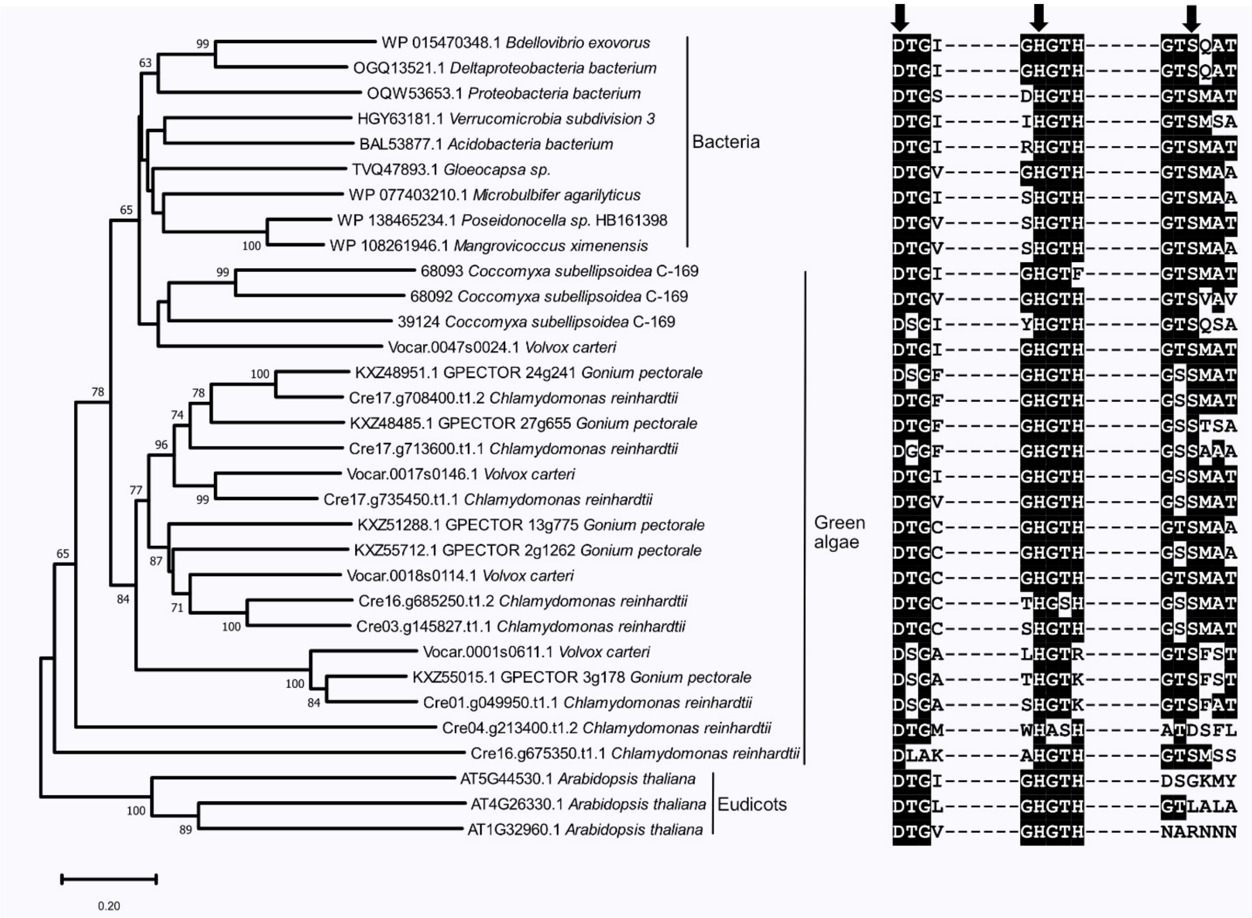

Supplemental Fig. S4 The phylogeny of sporangin (Cre04.g049950) and its close homologues from Chlamydomonas and bacteria. Three most similar subtilisins from Arabidopsis are used as outgroups. The phylogenetic analysis was performed using MEGA X as in Fig. 3. Only the bootstrap values above 50 are shown. The sequence motifs containing three conserved catalytic amino acids are aligned and active site residues are indicated by arrows.
